# A Randomized, Double-Blinded, Clinical Trial on Effects of a *Vitis vinifera* Extract on Cognitive Function in Healthy Older Adults

**DOI:** 10.3389/fphar.2017.00776

**Published:** 2017-10-31

**Authors:** Gioacchino Calapai, Francesco Bonina, Andrea Bonina, Luisa Rizza, Carmen Mannucci, Vincenzo Arcoraci, Germana Laganà, Angela Alibrandi, Concetta Pollicino, Santi Inferrera, Umberto Alecci

**Affiliations:** ^1^Department of Biomedical, Dental Sciences and Morphofunctional Imaging, University of Messina, Messina, Italy; ^2^Department of Drug Sciences, University of Catania, Catania, Italy; ^3^Bionap srl, R&D Contrada Fureria, Belpasso, Italy; ^4^Department of Clinical and Experimental Medicine, University of Messina, Messina, Italy; ^5^Italian College of General Practitioners and Primary Care, Florence, Italy; ^6^Department of Economics, Unit of Statistical and Mathematical Sciences, University of Messina, Messina, Italy

**Keywords:** *Vitis vinifera*, aging, cognitive decline, neuropsychological status, food supplement

## Abstract

**Introduction:** Gradual population aging is creating a new set of needs in the general population. Memory capacity decreases with age, and memory deficits are considered an early symptom of Alzheimer’s Disease (AD), one of the most prevalent cognitive disorders in older people. Numerous studies have shown that grape polyphenolic compounds (GPs) are able to attenuate cognitive impairment and reduce brain lesions in experimental AD animal models. These GP effects are associated with improvement in brain antioxidant status and prevention of free radical-induced neuronal damage. We designed a randomized, double-blind, placebo-controlled clinical trial to investigate the potential beneficial effects of a *Vitis vinifera-*based dietary supplement on cognitive function and neuropsychological status in healthy older adults.

**Methods:** One-hundred eleven subjects were recruited and randomly divided in two groups: one group received the *V. vinifera-*based dietary supplement Cognigrape^®^ for 12 weeks (250 mg/day) and the second group received placebo over the same period of time. Before and after the end of the supplementation period, cognitive function and neuropsychological status were evaluated using the Mini-Mental State Examination (MMSE), Beck Depression Inventory (BDI), Hamilton Anxiety Rating Scale (HARS), and Repeatable Battery for the Assessment of Neuropsychological Status (RBANS) evaluations.

**Results:** MMSE scores were significantly improved after supplementation with Cognigrape^®^ in comparison with baseline levels (*p* < 0.0001) and placebo (*r* = 0.59, 0.95% CI 0.11, 1.22; *p* < 0.0001). Cognigrape^®^ supplementation produced a significant reduction in BDI (-15.8%) and HARS (-24.9%) scores with respect to baseline levels (*p* < 0.0001) and placebo (*p* < 0.0001 for BDI and *p* < 0.05 for HARS). RBANS total score was significantly improved by Cognigrape^®^ with respect to baseline levels and placebo (*r* = 0.55, 0.95% CI 0.48, 6.07; *p* < 0.0001). The comparison with the placebo revealed improvements in several parameters among participants receiving Cognigrape^®^: attention (*p* < 0.001); language (*p* < 0.05); immediate memory (*p* < 0.0001); and delayed memory (*p* < 0.0001). Visuospatial/constructional abilities were not modified. During the study, no adverse effects were detected.

**Conclusion:** The results show that 12 weeks of Cognigrape^®^ supplementation is safe, can improve physiological cognitive profiles, and can concurrently ameliorate negative neuropsychological status in healthy older adults.

## Introduction

The increase in life expectancy is associated with gradual aging of the population. This epochal change creates new needs arising from this new situation. It has been observed that memory ability decreases with age, and memory deficits are considered an alarm signal because they are an early symptom of Alzheimer’s Disease (AD) which is one of the most prevalent cognitive disorders occurring in elderly people (Petersen et al., 2001; Vecchio et al., 2016). Prevalence estimates indicate that many older people (22.2%) show cognitive decline without dementia and that annually about of 12% of them will develop dementia (Plassman et al., 2008). The scientific community has to face this challenge and find effective strategies to prevent or slow the consequent physiological cognitive decline with the aim of preventing the increase in dementia.

Several methods have been suggested to support healthy aging. It has been proposed that polyphenolic compounds found in colored fruits and vegetables such as *Vitis vinifera* can contribute to improving memory and cognition during aging (Fuhrman et al., 2005). Research studies have demonstrated the effects of *V. vinifera*, including antioxidant, antibacterial, anti-inflammatory, anticancer, and antidiabetic activities, skin protection, and cardioprotective, hepatoprotective, neuroprotective properties (Nassiri-Asl and Hosseinzadeh, 2016). Among the chemicals contained in *V. vinifera*, its polyphenolic compounds have shown to be responsible for antioxidant, anti-inflammatory, antimicrobial, and antitumoral activities. These properties have facilitated a great interest in developing products based on these compounds in order to prevent or treat chronic and degenerative diseases, including neurodegenerative diseases (Wang et al., 2012). Numerous *in vivo* studies have demonstrated that orally administered polyphenolic compounds derived from multiple dietary sources, but more specifically grape polyphenolic compounds (GPs) were able to attenuate the cognitive impairment and in reducing neuropathological lesions in the brain in experimental animal models for the study of Alzheimer’s Disease (AD) as a result of their antioxidant activity (Rezai-Zadeh et al., 2005; Hartman et al., 2006).

Polyphenolic compounds and their derivatives are contained in GPs. These substances are synthesized by plants and stored to be used against mechanical damage, fungal attack, or ultraviolet rays (Frémont et al., 1999; Oliboni et al., 2011). It has been also observed that the GPs are able to reduce deposits of lipofuscin (a marker of lipid peroxidation) and significantly inhibit the depletion of ethanol-induced antioxidant enzymes in hippocampal brain regions involved in short-term spatial memory (Assuncao et al., 2007). GPs are a combination of proanthocyanidins, consisting of flavan-3-ol units such as catechin, catechin gallate, epicatechin, and epicatechin gallate, all of which represent the most abundant and complex polyphenols in grapes. These compounds form the basis of various types of polymers linked through C43C8 or C43C6 interflavan bonds (Sharma et al., 2011). In *V. vinifera*, proanthocyanidins exert a role as defense metabolites against pathogens and herbivores (Santos-Buelga and Scalbert, 2000). The intake of proanthocyanidins, especially in the monomeric form, have been shown to produce an improvement of cognitive function in an Alzheimer’s disorder animal model. This effect of proanthocyanidins is mediated via promotion of basal synaptic transmission in the hippocampal areas and the reduction in high molecular weight amyloid beta protein oligomer aggregation in the brain (Wang et al., 2009; He et al., 2016). In addition, scientific studies using *in vivo* behavioral tests (such as the Morris water maze test) were conducted to evaluate the beneficial effects from GPs on spatial learning and storage capacity. In these studies, a significant improvement in performance levels in animals fed with compounds containing GPs was observed (Shukitt-Hale et al., 2006).

In light of the above findings, our study highlights results on the effectiveness and safety of Cognigrape^®^, a food supplement ingredient based on a *V. vinifera* extract. We conducted a randomized, double-blind, placebo-controlled clinical trial. We hypothesized that extract intake could improve cognitive function and neuropsychological status in comparison to placebo intake in a sample consisting of healthy older individuals.

## Methods

### Study Design

The study was a randomized into a two-group, parallel, placebo-controlled clinical trial to assess the efficacy of a food supplement developed for cognitive and neuropsychological functioning in healthy older adults.

### Inclusion and Exclusion Criteria and Screening

Participants were recruited by 10 different general practitioners coordinated by a principal investigator according to several inclusion criteria: (1) age ≥55 and ≤75 years; (2) speaks and understands Italian; (3) no food supplements designed to affect for cognitive functioning for a period of 2 weeks before recruitment and during the study period; and (4) score ≥24 on the Mini-Mental State Examination (MMSE). The exclusion criteria consisted of several parameters: (1) age <55 or >75 years or (2) affected by AD or other related disorders, psychiatric or neurological diseases (including aphasia, sensory, motor or visual disturbances which could affect the test results), cancer, coagulation disorders, cardiovascular, lung, kidney, thyroid, liver, gastrointestinal disease or insulin-dependent diabetes, excessive consumption of alcohol or substance abuse/dependence. Other exclusion criteria included more than three medical hospitalizations within the last year, subjects taking coumadin, tricyclic antidepressants, antipsychotics, and/or anticonvulsants, or subjects already taking medications for cognitive functioning. Prior to study initiation, all subjects received oral and written information about the trial and gave individual written informed consent to participate after explanation of the aims and possible consequences of the study. A sample size of at least 51 participants per group was required to provide a power of 80% with α = 0.050 in order to detect 25% difference in improvement rate between treatment and placebo group.

The trial ran from the beginning of April 2016 to the end of April 2017 and was conducted according to the ethical principles of the Seoul revision (2008) of the Helsinki Declaration and Good Clinical Practice. The study protocol received approval by the Ethical Committee of the Azienda Policlinico Universitario “G. Martino” of Messina on March 10, 2016, protocol number 22/16 (ClinicalTrial.gov identifier: NCT3145987).

Mental status of eligible participants was evaluated through the MMSE, a test that is able to provide a quick screening for orientation, recall, language, orientation, registration, attention, and calculation (Folstein et al., 1975). The score is corrected for age and education, and the threshold for the purposes of enrollment in the study was set at a score ≥24 (Doody et al., 2001).

At the screening (visit 1, day 0), after giving informed consent, subjects provided their medical history, underwent a physical examination, and had vital signs monitored. After completion of those steps, subjects completed the MMSE. Out of 126 eligible participants, 111 healthy subjects (male/female ratio: 54/57) were enrolled.

**FIGURE 1:**
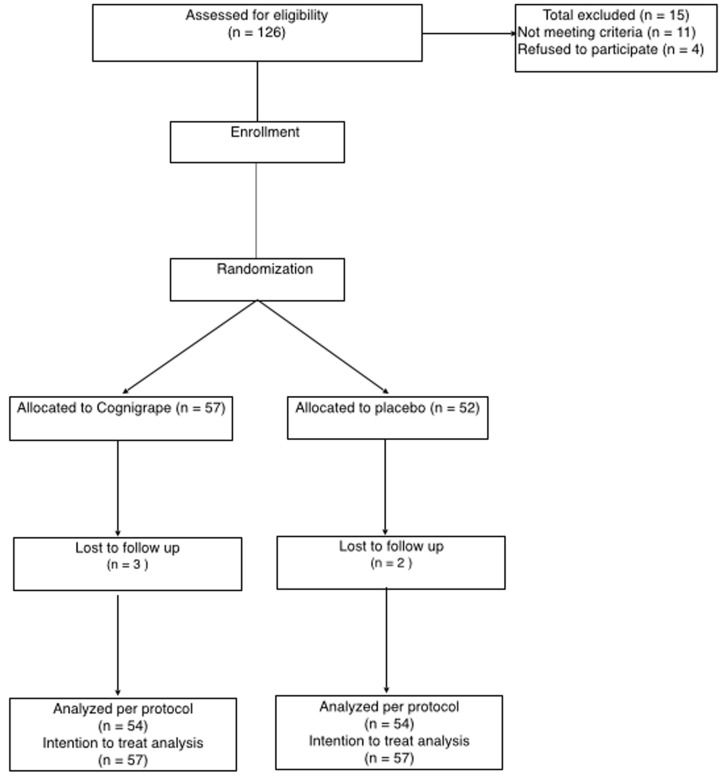
Study flowchart.

After screening and evaluation of MMSE score, included subjects were randomly allocated to one of two arm groups and treated according to the protocol: (1) the first group (N=57) was orally supplemented one time a day with 250 mg of Cognigrape^®^ (Bionap srl, Italy) and (2) second group (N=54) was orally supplemented one time a day with a placebo capsule. Randomization by blocks of 3 (2 + 1) was double-blinded. Successive blocks were balanced by 2 s. Each single Cognigrape^®^ capsule (250 mg) contained *V. vinifera* fruit extract and maltodextrin (30–40%). *V. vinifera* extract consisted of proanthocyanidins (>9% w/w) and anthocyanins as malvidin-3-glucoside (4–5% w/w) as verified by spectrophotometry. Placebo capsules did not contain *V. vinifera* extract and were composed only of maltodextrin. Neither recruited subjects nor investigators were able to differentiate between the two different treatments. The study flowchart is represented in **Figure 1**. Adverse events (AEs) were monitored during all the study period. An AE was defined as an inconvenient medical event occurring during the study period whether or not it was linked to the study procedure or the study product. A severe AE (SAE) was defined as a medical event resulting in death, a life-threatening situation, requiring inpatient admission or prolongation of hospitalization, and/or resulting in severe or persistent subject disability or incapacity.

### Assessment of Cognitive Functioning and Neuropsychological Status

Cognitive functioning and neuropsychological status were assessed by trained psychologists through the administration of several tests: (1) MMSE; (2) Beck Depression Inventory (BDI); (3) Hamilton Anxiety Rating Scale (HARS); and (4) Repeatable Battery for the Assessment of Neuropsychological Status (RBANS). All of the tests were administered by trained examiners before the beginning of the supplementation period and after 12 weeks (at the end of supplementation period) at least 18 h after the participants’ last supplemental dose. Thus, we were assessing whether chronic daily Cognigrape^®^ supplementation would elicit sustained cognitive and neuropsychological benefits. Participants were advised not to take any additional food supplements, modify eating habits, or change the levels of physical activity during the treatment period.

The MMSE (Folstein et al., 1975; Magni et al., 1996) is composed of 12 items consisting of verbal and performance trials and seven cognitive functions: (1) temporal orientation; (2) spatial orientation; (3) immediate memory; (4) attention and calculation; (5) recall memory; (6) language; (7) praxia visuo-constructive. An MMSE score between 18 and 24 is an indication of a compromised moderate to mild, while a score of 25 is considered borderline and from 26 to 30 is indicative of normality cognitive. For this reason, we chose a score limit of 24, which included masked potential light cognitive decline in healthy adults.

Beck Depression Inventory (Beck, 1967) is a self-report instrument that measures depression severity. We administered the BDI short form, which is a self-reported test for depression. It is a 13-item questionnaire that assesses four major components of depression: (1) behavioral; (2) affective; (3) cognitive; and (4) physiological components. Assigned values, indicating increasing severity, range from 0 to 3. According to Beck’s criteria, a score between 8 and 15 indicates moderate depression and >16 is indicative of severe depression (Seidel et al., 2015). The BDI questionnaire is composed of 21 questions with multiple choice responses that are suitable to investigate the presence of depressive symptoms.

The HARS (Hamilton, 1959; Andreescu et al., 2008) is a scale that evaluates anxiety through the investigation of 15 different areas (such as insomnia, mood, and somatic symptoms). Each of the 15 areas is composed of a minimum of three to a maximum of eight items, each of which is given a score from 0 to 6 depending on the severity of symptoms. Subsequently, the total value is calculated for each area using a score of 0 (absent), 1 (mild), 2 (moderate), 3 (severe), or 4 (very severe) points based on the overall severity of symptoms after investigating each specific area. The total score is calculated by adding the points of each of the 15 areas surveyed. The scale rating may vary from 0 to 56. A total score around 18 is considered indicative of a pathological state.

The RBANS is a quick and complete neuropsychological questionnaire initially designed as a tool to assess dementia, successively validated also for “normal” elder subjects and widely adopted for clinical diagnostic purposes and clinical studies. The RBANS produces index scores for five single neurocognitive domains and a Total Scale Index score. It is consisting of two forms (“A” and “B”) that are associated with identical difficulty degrees. Each is divided into 12 subtests that are administered in order to evaluate five different cognitive domains in five different time periods: (1) attention; (2) language; (3) visuospatial/constructional abilities; (4) immediate memory; and (5) delayed memory (Randolph et al., 1998; Novitski et al., 2012).

### Statistical Analysis

The numerical data were expressed as mean ± standard deviation (SD), and the categorical variables were expressed as number and percentage. The examined variables were not normally distributed, as verified by the *Kolmogorov–Smirnov test*. Consequently, a non-parametric approach was used. For each numerical parameter for basal observations and after 12 weeks, we separately performed statistical comparisons between treated and placebo patients using the *Mann–Whitney test*. For each numerical parameter, which were considered separately for treated and placebo patients, we performed comparisons between basal observations and those after 12 weeks in order to evaluate the existence of statistically significant differences in times; for this aim we applied the *Wilcoxon test*. The sample size was established considering an effect size of 0.25 in improvement rate between treatment and placebo group, a 2-sided significance level of 0.05 and a power of 80%. It was determined that 51 patients per group would be needed. Sample size was estimated for intention to treat analysis. Statistical analyses were performed using SPSS 21.0 for Window package. *P* < 0.05 two sided was considered to be statistically significant.

## Results

The mean age of the total sample of participants before randomization was 66.9 ± 5.2 years (range: 56–75 years, median age 68 years). Education levels of the total sample was reported as the mean of 11.25 ± 2.8 years. At baseline, both groups were found to be well matched for demographic characteristics (mean age, range of ages, median age, sex, body mass index, years of education) (**Table 1**) including daily physical activity; body mass index and daily physical activity have not changed during and at the end of the 12 weeks of the study.

**Table 1:** Baseline demographic characteristics of experimental (Cognigrape^®^) group and control (placebo) group of participants to the 12 weeks study.

	Experimental group *N* = 57	Control group *N* = 54	Statistics
Age, years (mean)	66.86 ± 5.3	66.90 ± 5.2	N.S.
Age range (years)	56–74	56–75	
Median age (years)	68	67.5	
Male/Female	27/30	26/28	
Body Mass Index	23.1 ± 1.1	23.3 ± 0.9	N.S.
Education (years)	11.22 ± 2.8	11.25 ± 2.9	N.S.

*N.S., not significant.*

**FIGURE 2:**
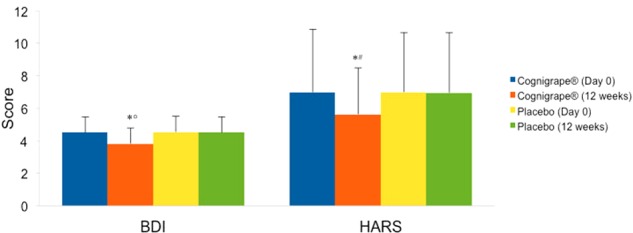
Effects of Cognigrape or placebo supplementation for 12 weeks on Beck Depression Inventory (BDI) and Hamilton Anxiety Rating Scale (IIARS) scores in healthy older adults. ^∗^*p* < 0.0001 vs. Cognigrape (Day 0); °*p* < 0.0001 vs. Placebo (12 weeks): ^#^*p* < 0.05 vs. Placebo (12 weeks).

**FIGURE 3:**
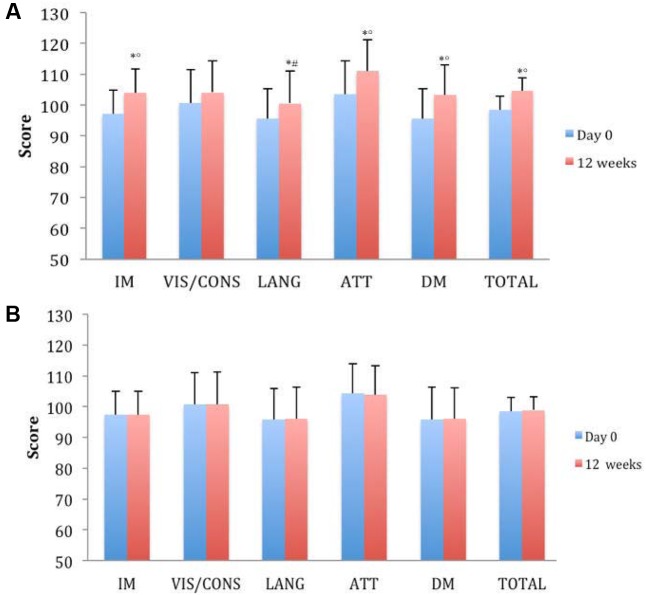
Effects of Cognigrape **(A)** or placebo supplementation **(B)** for 12 weeks on Repeatable Battery for the Assessment of Neuropsychological Status (RBANS) in healthy older subjects. IM, immediate memory; VIS/CONS, visuospatial/constructional abilities; LANG, language; ATT, attention; DM, delayed memory. ^∗^*p* < 0.01 vs. Cognigrape (Day 0); °*p* < 0.01 vs. Placebo (12 weeks); ^#^*p* < 0.05 vs. Placebo (12 weeks).

Mini-Mental State Examination baseline score was significantly improved only in the group treated with Cognigrape^®^ for 12 weeks (*p* < 0.0001). MMSE improvement obtained with Cognigrape^®^ was also statistically significant also when compared to placebo group (*r* = 0.59, 0.95% CI 0.11, 1.22; *p* < 0.0001). These results and the mean changes in MMSE scores are shown in **Table 2**. No significant effects resulting from gender were found in either group regarding differences in MMSE, BDI, HARS, and RBANS scores from baseline. In **Figure 2**, the baseline and final BDI and HARS mean scores and standard deviations of groups receiving Cognigrape^®^ or placebo supplementation for 12 weeks are shown. BDI baseline mean scores of the total sample were 4.52 ± 0.97. Total BDI mean scores and BDI mean scores of the two groups of subjects overlapped with reported epidemiological distribution of BDI mean scores in healthy adults (Segal et al., 2008); total scores from all subjects were under the level indicating depression. The results of the subjects’ BDI answers among those treated with Cognigrape^®^ showed a statistically significant decrease in the percentage (-15.8%) of the BDI score with respect to baseline (*p* < 0.0001). Similarly, a significant reduction was observed when Cognigrape^®^ BDI results were compared to placebo (*p* < 0.0001). In HARS, the mean score for the 111 participants was 6.97 ± 3.8. Cognigrape^®^ supplementation significantly reduced HARS baseline score by 24.9%, and this reduction was significant in comparison to placebo group (*p* < 0.05); all subjects’ total scores under this level indicated a pathological state of anxiety (**Figure 2**). RBANS total score was significantly increased by Cognigrape with respect to baseline levels and placebo (*r* = 0.55, 0.95% CI 0.48, 6.07; *p* < 0.001) (**Table 3**). Among the RBANS items, Cognigrape supplementation compared to placebo group significantly influenced several parameters items: (1) attention (*p* < 0.001); (2) language (*p* < 0.05); (3) immediate (*p* < 0.0001); and (4) delayed (*p* < 0.0001). Memory. RBANS scores of Cognigrape^®^ and placebo groups related to visuospatial/constructional abilities did not show any statistical differences (**Figure 3**). During the study period, no AEs were detected.

**Table 2:** Mini Mental State Examination (MMSE) score before (Day 0) and after supplementation with Cognigrape or Placebo for 12 weeks.

Supplementation	MMSE score (Day 0)	MMSE score (12 weeks)	Change in score
Placebo	27.21 ± 1.32	27.32 ± 1.26	0.11 ± 0.32
Cognigrape^®^ (250 mg/day)	27.24 ± 1.32	28.50 ± 1.01^∗∘^	1.22 ± 0.66^#^

*^∗^*p* < 0.0001 vs. Cognigrape^®^ Day 0; °*p* < 0.0001 vs. Placebo 12 weeks; ^#^*p* < 0.01 vs. Placebo (Change in score).*

**Table 3:** Effects of Cognigrape or placebo supplementation for 12 weeks on Repeatable Battery for the Assessment of Neuropsychological Status (RBANS) total score in healthy older subjects.

Supplementation	RBANS total score (Day 0)	RBANS total score (12 weeks)	Change in score
Placebo	98.57 ± 4.37	98.84 ± 4.35	0.48 ± 5.30
Cognigrape (250 mg/day)	98.50 ± 4.36	104.62 ± 4.31^∗∘^	6.07 ± 1.05^#^

*^∗^*p* < 0.001 vs. Cognigrape RBANS total score (Day 0); °*p* < 0.0001 vs. Placebo RBANS total score (12 weeks); ^#^*p* < 0.001 vs. Placebo change in score.*

## Discussion

This study examined the cognitive profile in healthy older adults following the intake of an ingredient (Cognigrape^®^) that is based on a *V, vinifera* extract or placebo for 12 weeks. Contextually, some aspects of neuropsychological status of subjects enrolled for the study were assessed. The main findings of the study indicated that cognitive performance in addition to neuropsychological status significantly improved at the end of the 12-week supplementation period in the group of participants who had taken Cognigrape^®^ in comparison to those who had taken placebo.

One important point of the study demonstrates for the first time that an improvement of cognitive performance following *V. vinifera* extract supplementation in humans occurs. This finding is in agreement with previous pre-clinical studies that have investigated grape cognitive effects. These studies suggested that chronic treatment with polyphenolic preparations from grapes result in the increase in brain bioactive metabolites that are able to counteract pathological mechanisms and restore neuronal function associated with learning and memory processes (Wang et al., 2012). Other *in vitro* findings have shown that GPs extracts can significantly reduce aggregation of amyloid β-protein into high-molecular weight oligomers, which are considered responsible for dementia and memory loss in AD. The same authors also demonstrated that oral administration of a GPs preparation to Tg2576 mice, an experimental model used to study AD, significantly attenuated AD-type cognitive deterioration concurrently with a reduction in high-molecular weight soluble oligomeric brain Aβ, thus suggesting that GP polyphenolics may be useful agents for AD prevention or treatment. Another conceivable mechanism for GPs’ protective effects occurs through antioxidant activity that could maintain oxidative balance, thus preventing oxidative repression of brain-derived neurotrophic factor (Wang et al., 2008).

It is known that reduction of the antioxidant capacity, together with diminished brain-derived neurotrophic factor levels, can be the responsible for behavioral impairments (Okuno et al., 2011). Other experimental studies suggested that grape protective role could be due to epigenetic regulation of brain derived growth factor (Solanki et al., 2015) or to the prevention of the stress-induced decline of antioxidant enzymes as well as of brain-derived neurotrophic factor, in specific brain regions, including the hippocampus (Allam et al., 2013; Patki et al., 2013).

Among polyphenols, flavonoid anthocyanidins are the most abundant pigments contained in grape skin. Their conjugated derivatives, anthocyanins, pigments conferring the color hues of blue, red, and purple to fruits, flowers, and plant organs (Clifford, 2000) and proanthocyanidins, also known as condensed tannins (Santos-Buelga and Scalbert, 2000) are present in significant concentrations in *V. vinifera* extracts used in this study. It can thus be hypothesized that they are responsible for the positive effects on cognitive profiles of healthy older adults that we observed in the present study.

In our study, as shown by baseline scores, the intake of the *V. vinifera* extract reduced the BDI score in healthy subjects who were not affected by depression. Reduction in the BDI score suggests an improvement in mood that can be associated with positive effects, thus producing a better cognitive performance.

Reduction of HARS score, associated with improvement of cognitive performance evaluated with MMSE and RBANS in subjects receiving supplementation with *V. vinifera* extract, confirms a relationship between reduction of cognition and potential state anxiety. Our data are according to previous research linking anxiety with cognitive impairment and confirm that even within normal limits, potential state of anxiety can be associated with poorer global cognitive functioning (Manela et al., 1996; Beekman et al., 1998). People recruited for the study did not show baseline BDI or HARS score levels indicating a pathological state of depression or anxiety; this finding could explain the lack of effects of placebo supplementation in reducing both the scores. It is possible to hypothesize that supplementation with the *V. vinifera* extract ameliorated the cognitive profile positive influences and also psychological status since we observed a reduction in BDI and HARS scores. Studies on the relationship of anxiety with cognitive ability present evidence of increasing anxiety in persons with cognitive dysfunction (Johnsen and Asbjørnsen, 2009; Haikal and Hong, 2010). Moreover, it has been also observed that older subjects showing impairment of short-term and delayed memory frequently become anxious people (Mantella et al., 2007). On this basis, it is possible to hypothesize that improvement of cognitive performance detected by intake of *V. vinifera* extract contributed to reduction in pre-anxiety score levels in our study.

It is known that aging is associated with physiological cognitive decline and changes in emotional status, and that impairment in memory and cognitive function is a normal consequence of aging (Bishop et al., 2010). Interventions to prevent decline of cognitive function must be aimed at memory restoration and strengthening of cognitive skills in order to preserve function in the everyday context. Grape intake is commonly considered to promote good health, and its effects have been associated with mental well-being (Thomas et al., 2009; Wang et al., 2009; Vislocky and Fernandez, 2013). In particular, grapes’ antioxidant properties have been attributed predominantly to the content of numerous polyphenolic constituents, largely believed to be responsible for their beneficial effects (Patki et al., 2015). However, anti-inflammatory and antiapoptotic mechanisms have also been proposed to explain protective effects (Yousuf et al., 2009; Salim et al., 2011). Our data show improvements in some aspects of cognitive functioning such as attention, memory, and language in healthy adults after supplementation with a *V. vinifera*-based ingredient. The *V. vinifera* extract used in the current study was well-tolerated, as shown by the lack of AEs. The results of this clinical trial confirm previous pre-clinical findings suggesting potential neuroprotective effects of grapes and provide evidence for a beneficial effect of supplementation with a *V. vinifera* extract on cognitive function in healthy older adults. Improvements in cognitive profiles are consistent with prior investigations on antioxidant activity of proanthocyanidins and anthocyanins contained in *V. vinifera* and retained in the commercial formula. This *V. vinifera-*based formula has been shown to be safe, and its utilization may produce cognitive improvements associated with neuropsychological benefits in healthy subjects.

## Author Contributions

GC: Design and coordination of the study, interpretation of results and writing. FB and AB: Development and characterization of the study product. LR: Development and characterization of the study product and writing. CM: Design and monitoring of the study and writing. GL and SI: Recruitment of participants, product administration, medical visits and follow up. VA: Interpretation of results. AA: Statistical analysis. CP: Cognitive test administration and interpretation. UA: Recruitment of participants, product administration, medical visits, follow up, interpretation of results.

## Conflict of Interest Statement

The authors declare that the research was conducted in the absence of any commercial or financial relationships that could be construed as a potential conflict of interest.
